# Graves' Disease

**Published:** 1895-11-09

**Authors:** 


### Progress in Medicine.

### GRAVES' DISEASE (*Continued*).

**Ligature of Arteries.**—Ligature of all the arteries has been performed by Trondelbenberg[59] in two sittings at an interval of a few weeks, without myxœdematous symptoms arising. Rydygler's[60] experience in 22 cases is the same in this respect. An interesting fact is related by Kocher,[57] viz., that in his six cases of extirpation of goitre for Graves' disease three were fatal, whereas in 870 cases of extirpation for non-malignant goitres he had only had three deaths.

**Thyroid Injections.**—The effect of thyroid injections in Graves' disease has already been referred to in considering the recent literature bearing on the pathology of Graves' disease, but it will be convenient to include these cases again in the following notes. Kocher[61] believes that the favourable effect of thyroid extract in Graves' disease may be explained by the fact that acute cachexy, with symptoms of tetanus, supervened in some of his cases in the first few hours following extirpation of a goitre or ligature of the thyroid arteries, suggesting auto-intoxication by retention of a toxic substance formed in the nervous system itself or in some other organ. It is known that these symptoms rapidly yield to ingestion of thyroid juice, which thus appears to neutralise the toxic substance in question. This led him to suggest an abnormal irritation of certain parts of the nervous system by toxic products formed in the latter or in some other organ, capable of determining hyperplasia of the thyroid gland, which is called upon to furnish in larger quantities the neutralising substance. Hyerplasia of the thyroid, then, constitutes a means of defence on the part of the nervous system against injurious action of certain toxic substances elaborated in the organism. Later on, in consequence of degeration in the gland, symptoms of myxœdema supervene.

Several observers have employed thyroid extract without improvement, viz., Madison, Taylor,[62] Joffroy,[63] while Voisin[64] reports excellent results in two cases (four grammes of gland daily), and favourable results are reported by Bruns, Reinhold, Béclere, and Bogrof. Voison believes that impaired quality of the thyroid secretion is the principal cause of the disease. Thyroid feeding doubtless destroys the toxins which the diseased thyroid gland throws into the circulation, or else it must be assumed that the ingested substance makes up for the insufficiency of the secretion of the diseased gland. He believes in the former hypothesis, for in one of his patients, in whom the thyroid treatment was irregularly administered, diarrhœa, emotivity, tremors, and palpitation reappeared within a few days after suspension of the treatment, and disappeared again two days after its resumption.

**Electricity.**—We have referred to cases recorded by Tuffier[51] and McLosh,[48] in which electrical treatment had no effect. Bordeer[65] reports two cases treated with much benefit. The applications (Faradaic) by Vigouroux's method were: (1) To the upper branch of the facial nerve and to the orbicularis palpebrarum, half a minute to each side; (2) to the neck between the hyoid bone and anterior border of the sterno-mastoid, the electrode pressed deeply over the carotid artery, one and a half minute each side; (3) over the thyroid swelling for five minutes; (4) over the precordium for three minutes. The thyroid muscles were also made to contract a few times at each sitting. Treatment three times weekly. Indifferent electrode applied to the nape of the neck throughout. Boyd[66] records a case in a man, age 32, who, after suffering from anorexia, repeated bilious attacks and impaired digestion, developed Graves' disease, large thyroid, proptosis, pulse 150, and marked excitability. The treatment consisted in iodide, hydriotic acid, digitalis, galvanism daily over the thyroid, and rest, physical and mental. At times ice-bags to the thyroid when the excitement was very great. Result—improvement in every respect, proptosis greatly diminished, pulse 80-90, no excitement, and thyroid reduced; he was able to resume work. Joffroy[67] strongly recommends either galvanism or faradism, the negative pole applied to the heart, belly, and thyroid, while the positive is applied to the nape of the neck.

**Climate.**—Glax[68] mentions five cases greatly improved by the mild sea climate of Abbazia. He remarks that this very favourable action of sea air has previously been noted in Graves' disease.

**Drugs.**—Belladonna is rejected by Joffroy[69] as unreliable, but he believes in the use of strophanthus, bromide of potassium, and antipyrine. The application of ice and of cold douches to the thyroid is commended by Joffroy, Boyd,[70] and Fridenberg.[71] The two latter practitioners also advocate digitalis; Joffroy prefers strophanthus; Chibert[72] speaks of good results obtained by him with salicylate of soda. In each of four cases marked improvement took place in a few days. In others the symptoms returned on suspending the drug. The writer was led to try this dıug in the first instance by finding a family arthritic history. In one case, if the patient was over-fatigued or chilled, the salicylate, which had been discontinued after a lengthy period of improvement, was resumed to prevent return of the symptoms. Trachewsky has found that sodium phosphate (three to four grammes daily) causes marked improvement in cases of Graves' disease.

The effects of the administration of thymus gland have already been related.

[58] Med. Week, Apr. 26, 1895. [59] Ibid. [60] Ibid. [61] Med. Week, Mar. 1, 1895. [62] N. Y. Med. Jour., Nov. 24, 1894. [63] Med. Week, 1895, p. 393. [64] Ibid. [65] Ther. Gaz., Mar. 15, 1895; Arch. d'Elect. Méd., Oct., 1894. [66] Med. Rec., Mar. 30, 1895. [67] Prog. Méd. XIX., 13, 1894. [68] Wien. Med. Woch., Mar. 2, 1895; B. M. J. Supp., Apr. 6, 1895. [69] Progrès. Med. XIX., No. 13, 1894. [70] Loc. cit. [71] Med. Rec., July 13, 1895. [72] Journ. de Méd., Apr. 10, 1895; Sup. B. M. J., June 8.

